# Interactions between glutathione S-transferase genes and household air pollution on asthma and lung function

**DOI:** 10.3389/fmolb.2022.955193

**Published:** 2022-09-29

**Authors:** Xin Dai, Shyamali C. Dharmage, Caroline J. Lodge

**Affiliations:** Allergy and Lung Health Unit, Centre for Epidemiology and Biostatistics, Melbourne School of Population and Global Health, University of Melbourne, Melbourne, VIC, Australia

**Keywords:** review, lung function, asthma, household air pollution, glutathione S-transferase

## Abstract

Oxidative stress is one of the main pathophysiological mechanisms for chronic respiratory disease. Glutathione S-transferase (GST) genes play important roles in antioxidant defences and may influence respiratory health. Although there is not consistent evidence that the three commonly studied genes of *GSTM1*, *GSTT1* and *GSTP1* are associated directly with respiratory outcomes, they seem to be related to disease susceptibility if exposure interactions are taken into account. Exposure to household air pollution may be particularly important in increasing lung oxidative stress. This review summarizes the relationships between GST genes, household air pollution and asthma and impaired lung function. Our findings support a role for GST polymorphisms in susceptibility to asthma and impaired lung function via oxidative stress pathways. Future research should additionally consider the role of gene-gene interactions, multiple environmental exposures, and gender in these complex associations, that are involved in maintaining antioxidant defences and lung health.

## Introduction

Over the past 20 years, the prevalence of asthma has been increasing significantly worldwide, particularly for countries transitioning to a developed economic status (Global Asthma Network, 2014). Asthma is not a single disease but rather an umbrella term for multiple phenotypes with similar clinical features including airway inflammation and reversible airway obstruction. In the longer-term asthma may lead to reduced lung function. Many people with chronic and/or severe asthma experience an accelerated and progressive loss of lung function over time and their lung function progresses from reversible to irreversible airway obstruction (Pascual and Peters, 2005).

Although the aetiology of asthma is unknown, many authors have found evidence that asthma is associated with increased oxidative stress in the airways; evidenced by the presence of increased oxidative biomarkers (Milos et al., 2017). Many air pollutants generate endogenous reactive oxygen species (ROS) that cause oxidant injury in the lungs. Detoxification is a primary method of defence from harmful environmental exposures. Glutathione S-Transferases (GSTs) play a major protective antioxidant role in the lungs and subsequently regulate inflammatory responses (Piacentini et al., 2013). GSTs catalyze the conjugation of antioxidants - glutathione (GSH) with electrophilic compounds that generate free radicals, thus leading to detoxifying effects. Polymorphisms of genes encoding GST enzymes are therefore crucial to regulating the detoxification of a wide range of endogenous compounds (Piacentini et al., 2013).

Findings on the relationship between household air pollution exposures and respiratory health outcomes have been inconsistent. A reason for the inconsistent findings between common oxidative household exposures and respiratory health could be the failure to account for potential gene-environment interactions. Over the last decade, there has been increasing evidence to suggest that gene-environment interactions are likely to be involved in the development, severity, and age-of-onset of many complex diseases including asthma (Minelli et al., 2011; Minelli and Gögele, 2011). It is possible that individuals with risk variants of GST genes may be more susceptible to environmental risk factors and more likely to develop asthma, compared to those without risk genes, even when exposed to the same environmental factors. However, there are few studies on GST gene-environment interactions and their associations with asthma and lung function and their findings are inconsistent. This review will discuss the three most researched classes of GST gene polymorphisms including *pi* 1 (*GSTP1*), *mu* 1 (*GSTM1*) and *theta* 1 (*GSTT1*), and summarize current knowledge on of complex mechanisms that may be involved in the interactions between household air pollution exposure and GST gene polymorphisms and potentially the aetiology of asthma in high risk populations who carry these risk alleles.

## Overview of glutathione S-transferase genes

Eight classes of the most studied GST gene superfamily code for similarly named glutathione transferases: *alpha, kappa, mu, pi, sigma, theta, omega* and *zeta* have been described (Table 1). Each of these classes are found in many different tissues and have multiple isoforms (Buratti et al., 2021). (Alexander et al., 2013). Of these, the three most extensively studied are pi 1, mu 1 and theta 1. These are also the classes that have been found to be expressed in the lung. The *GSTP1* gene is located on a ‘hot spot’ site for asthma-related genes - chromosome 11q13. The *GSTP1* enzyme is the most common form of GST expressed in lung epithelium, responsible for over 90% of lung GST enzyme activity (Minelli et al., 2011). The *GSTM1* gene spans a 97-kb region on chromosome 1p13 and is predominately expressed in the liver (Murdzoska et al., 2010). The *GSTT1* gene is located on chromosome 22q11 and predominately expressed in erythrocytes (Murdzoska et al., 2010). However, both *GSTM1* and *GSTT1* are also expressed in lung tissue, specifically in the bronchial wall (Breton et al., 2009). Polymorphisms of all these GST genes may influence antioxidant function in the lungs and other organs, and this can confer genetic susceptibility to oxidative stress and disease like asthma. Given polymorphisms of GST genes are common in the general population, there is public health importance of studying GST susceptibility profiles (Table 1). A common polymorphism in the *GSTP1* gene is Ile105Val, with the frequency of the Val105 allele lower in Asian and higher in European and African populations. The Val allele has been associated with higher risk of respiratory issues (Minelli et al., 2010). The frequency of absence polymorphisms for *GSTM1/T1* also varies across ethnic groups. (From 15 to 62% in specific populations) The null genotype of *GSTM1/T1* results in loss of enzyme activity and thus reduction of antioxidant capacity (Minelli et al., 2010). The variation of frequency of GST polymorphisms across ethnicities suggests a potential role of ethnicity in explaining the heterogeneity of associations found in the current literature.

**TABLE 1:** Genetic profiles for eight human GSTs.

Class	Isoforms	Chromosome location	Tissues expressed in (ordered from most to least)	Prevalence in general population
Alpha	GSTA1, GSTA2, GSTA3, GSTA4	6p12	testis, liver, kidney, adrenal, pancreas	GSTA1*B (low protein level) frequency: 16% in Asian, 40–42% in Caucasian, 35% in African Pascual and Peters. (2005).
Kappa	GSTK1	7q34	liver	A transition of G to T in -1308 SNP relative to the GSTK1 with a T allele frequency of approximately 0.20 in Chinese. No data in Europeans and Africans Milos et al. (2017).
Pi	GSTP1	11q13	Brain, heart, lung, testis, kidney, pancreas	Val105 frequency: 8–33% in Asians, 30–37% in Europeans, 14–53% in Africans Piacentini et al. (2013).
Mu	GSTM1, GSTM2, GSTM3, GSTM4, GSTM5	1p13	Liver, testis, brain, adrenal, kidney, lung	GSTM1 Null frequency: 32–53% in Asians, 35–62% in Europeans, 23–41% for Africans Minelli and Gögele. (2011).
Sigma	GSTS1	4q21-22	Fetal liver, bone marrow	Polymorphisms have not been reported Global Asthma Network (2014) Minelli et al. (2011).
Theta	GSTT1, GSTT2	22q11	Kidney, liver, small intestine, brain, prostate, lung	GSTT1 Null frequency: 38–58% in Asians, 15–31% in Europeans, 22–29% in Africans Minelli and Gögele (2011).
Zeta	GSTZ1	14q24	Fetal liver, skeletal muscle	Lys32 frequency: 49% in Chinese, 28–33% in Caucasian (Buratti et al., 2021). No Data in Africans.
Omega	GSTO1	10q23-25	Liver, heart, skeletal muscle, pancreas, kidney	Ala140 frequency: 84% in Asian, 67% in Caucasian, 92% in African. Alexander et al. (2013)

## Oxidative stress in the lungs and its relationship with asthma

Oxidative stress reflects the imbalance between the overproduction of oxidants in terms of ROS and reactive nitrogen species (RNS) and underproduction of antioxidant defence mechanisms in the body (Milos et al., 2017). There are many exogenous sources of ROS, such as cigarette smoke and air pollutants. These ROS play an important role in the pathogenesis of inflammatory conditions. After exposure to environmental triggers, normal physiological antioxidant defence mechanisms are usually able to eliminate ROS and repair damage. However, an imbalance between oxidants and antioxidants can have significant functional and structural consequences associated with excessive airway inflammation and without sufficient repair may lead to accumulated oxidative changes until this process reaches the “threshold” for lung damage and manifests as asthma (Milos et al., 2017). This mechanism is a long-term continuous and dynamic process and may explain the development and persistence of asthma (Milos et al., 2017).

The lungs are equipped with a range of antioxidant enzymes for managing the constant exposure to a wide range of oxidants. These enzymes include superoxide dismutase (SOD) and glutathione peroxidase Decreased activity or dysfunction of normal antioxidant enzyme defences can be identified in asthmatic airways. GSH is an important antioxidant substance in humans which can be found in the alveolar epithelial lining fluid and it protects against airway peroxidation (Fernando, 2013). People with mild asthma have been shown to have a high glutathione level, reflecting an adaptive response to increased ROS, compared to healthy people. However, in severe asthma, GSH levels have been shown to be significantly lower when compared to people with mild to moderate asthma or healthy controls. This may due to key enzymes involved in glutathione synthesis being highly dysfunctional (Fernando, 2013). The reduced GSH level may impair the ability to detoxify ROS.

GSTs can facilitate detoxification through both GSH dependant and GSH independent pathways. Firstly, GSTs can work in conjunction with GSH. During an antioxidative response, GSTs act as functional enzymes that catalyze the conjugation of GSH to several electrophilic compounds, these compounds are generally less reactive and more soluble. GSTs assist in the transport and elimination of conjugated oxidants by moving these compounds from cells via membrane-based GSH conjugate pumps (Martos-Maldonado et al., 2015). Secondly, GSTs also perform a large array of non-enzymatic functions, including inactivating compounds in the stress-response signalling pathways of the detoxification process (Hayes and Strange, 2000; Martos-Maldonado et al., 2015). GSTs can bind numerous ligands for cell surface receptors, thus they may block signalling to inflammatory compounds like prostaglandins (Hayes and Strange, 2000). Additionally, GST genes regulate a complex network of antioxidant defences involving many other antioxidant compounds such as arginine, citrulline, taurine, creatine, selenium, zinc and vitamins A, C, D and E, and antioxidant enzymes (Minelli and Gögele, 2011). Thus, genetic variations in GSTs are of interest in the study of disease susceptibility, as they may alter the influence of many household air exposures on respiratory health through oxidative stress and inflammatory pathways.

## Can glutathione S-transferase alone affect risk of asthma and lung function?

There is a strong biological rationale that GST polymorphisms may influence risk of asthma, and airway hyperresponsiveness via oxidative stress pathways (Lenney and Fryer, 2007), but the current evidence does not fully support this hypothesis (Table 2). No cohort study has demonstrated a main effect of GST genes on asthma outcomes, including two cohorts with large sample sizes (>2000 participants) and long-term follow up (Gilliland et al., 2002; Imboden et al., 2007). However, both these cohorts identified slower lung function growth/quicker lung function decline in populations with susceptible genotypes (Gilliland et al., 2002; Imboden et al., 2007). This discrepancy in findings may be partly due to the outcome of lung function, as a continuous variable, having greater power to detect associations compared to asthma outcomes (binary variable).

**TABLE 2:** Associations between GST genes and asthma and lung function.

References	Outcomes	Outcome age	Main results
Cohort studies			
Gilliland et al. (2002) Murdzoska et al. (2010)	Lung function growth	Children	GSTM1 null and GSTP1 Val/Val were associated with slower lung function growth in FEV1 and FVC. No evidence for GSTT1.
Imboden et al. (2007) Breton et al. (2009)	Self-reported asthma and lung function decline	Adults	No evidence for GSTM1, GSTT1 and GSTP1 for asthma outcomes.
			GSTT1 null genotypes alone or in combination with GSTM1 null were associated excess decline in FEV_1_ in men, not in women. No evidence for GSTM1 and GSTP1.
Schroer et al. (2009) Minelli et al. (2010)	Parent-reported wheezing	Children	No evidence for GSTP1.
Case-control studies			
Fryer et al. (2000) Fernando (2013)	Atopic asthma	Adults	GSTP1 with Val/Val alleles associated with reduced risk of asthma compared to Ile/Ile (OR 0.16, 95%CI 0.05, 0.55); No evidence for GSTM1 and GSTT1.
Chung (2002) Martos-Maldonado et al. (2015)	Physician diagnosed asthma	Adults	No evidence for GSTM1, GSTT1, or GSTP1.
Sideleva et al. (2002) Hayes and Strange (2000)	Atopic asthma	Adults and children	GSTM1 (OR 3.49, 95%CI 1.91, 6.38) and GSTT1 (OR 6.66, 95% CI 3.55, 12.52) null genotypes were associated with risk of asthma.
Vavilin et al. (2002) Lenney and Fryer (2007)	Physician diagnosed asthma	Children	No evidence for GSTM1 or GSTT1.
Safronova et al. (2003) Gilliland et al. (2002)	Bronchial asthma	Children	No evidence for GSTP1.
Aynacioglu et al. (2004) Imboden et al. (2007)	Physician diagnosed asthma	Adults	GSTP1 with Val/Val alleles were associated with decreased risk of asthma compared to those with Ile/Ile (OR 0.31, 95%CI 0.14, 0.69).
Mostafa et al. (2004) Tamer et al., (2004)	Lung function defined asthma	Adults	GSTM1 null genotypes (OR 3.17, 95%CI 1.65, 6.12) and GSTT1 null genotypes (OR 2.62, 95%CI 1.41, 4.87) were associated with increased risk of asthma
Saadat et al. (2004) Anthony A et al. (2000)	Physician diagnosed asthma	Adults and children	GSTM1 (OR 3.17, 95%CI 1.65, 6.12) and GSTT1 (OR 2.62, 95%CI 1.41, 4.87) null genotypes were associated with risk of asthma.
Tamer et al. (2004) Renate et al. (2005)	Physician diagnosed asthma	Adults	GSTM1 null genotypes (OR 2.51, 95%CI 1.43, 4.42) and GSTP1 with Val/Val alleles (OR 4.27, 95%CI 1.71, 10.69) were associated with increased risk of asthma; No evidence for GSTT1.
Zhang et al. (2004) Chung (2002)	Physician diagnosed asthma	Adults	GSTM1 (OR 10.39, 95% CI 4.42, 24.46) and GSTT1 (OR 19.15, 95%CI 7.28, 50.41) null genotypes were associated with increased risk of asthma.
Lee et al. (2005) Safronova et al. (2003)	Parent reported asthma	Children	GSTP1 with Ile/Val genotypes (OR 0.51, 95%CI 0.28, 0.93) were associated with decreased risk of asthma compared to those with Ile/Ile; No evidence for GSTM1.
Nickel et al. (2005)	Physician diagnosed asthma	Children	No evidence for GSTP1.
Oh et al. (2005)	Physician diagnosed asthma	Adults and children	No evidence for GSTP1.
Arbag et al. (2006) Ercan et al. (2006)	Physician diagnosed asthma	Adults and children	No evidence for GSTM1 and GSTT1.
Ercan et al. (2006) Vavilin et al. (2002)	Physician diagnosed asthma	Children	No evidence for GSTM1, GSTT1, or GSTP1.
Holla et al. (2006) Mostafa et al. (2004)	Physician diagnosed asthma	Adults and children	No evidence for GSTM1 or GSTT1.
	Allergic diseases		
Abdel-Alim et al. (2007) Zhang et al. (2004)	Physician diagnosed Asthma	Children	GSTP1 Val/Val was associated with decreased risk of asthma compared to Ile/Ile (OR: 0.14, 95% 0.05, 0.36).
Hanene et al. (2007)	Asthma (not. defined further)	Children	GSTM1 null genotypes (OR: 2.35, 95% CI 1.48, 3.75) were associated with increased risk of asthma, GSTP1 with Val/Val alleles (OR: 0.39, 95%CI 0.19, 0.80) were associated with decreased risk of asthma compared with those with Ile/Ile genotypes. No evidence of GSTT1.
Kamada et al. (2007) a Karam et al. (2012)	Physician diagnosed asthma	Adults and children	No evidence for GSTM1.
Kamada et al. (2007) b Karam et al. (2012)	Physician diagnosed asthma	Children	No evidence for GSTM1.
Mak et al. (2007) Iorio et al. (2015)	Physician diagnosed asthma	Adults	No evidence for GSTM1, GSTT1 or GSTP1.
Babusikova et al. (2009) Imboden et al. (2008)	Physician diagnosed asthma	Children	No evidence for GSTT1.
Castro-Giner et al. (2009) Frank D et al. (2002)	Self-reported asthma	Adults	No evidence for GSTM1, GSTT1 or GSTP1.
Mahmoud et al. (2011) Carr et al. (2004)	GINA criteria of asthma	Adults	No evidence for GSTP1.
Tatarskyy et al. (2011) Wu et al. (2014)	Physician diagnosed asthma	Adults and children	GSTP1 with any Val genotypes (OR 0.39, 95% CI 0.20, 0.77) was associated with decreased risk of asthma compared to Ile/Ile genotypes; No evidence for GSTM1 and GSTT1.
Turner et al. (2018) Lee et al. (2007)	Asthma attack	Children	No evidence for GSTM1, GSTT1 and GSTP1.
Karam et al. (2012) Wang et al. (2011)	Lung function and atopic asthma	children	GSTM1 null and GSTP1 Ile/Ile were associated with significant decreased in FEV1 and FVC compared to GSTM1 present and GSTP1 Val/Val genotypes. No evidence for GSTT1 for lung function.
			GSTM1 null was associated with increased risk of atopic asthma (OR 2.6, 95% CI 1.1, 6.4). No evidence for GSTT1 and GSTP1 on atopic asthma.
Dar et al. (2017) Hersoug et al. (2012)	Allergic asthma	Children	No evidence for GSTM1 and GSTT1.
Cross-sectional studies			
Salam et al. (2007) Dai et al. (2019)	Parent-reported asthma	Children	Marginal association between GSTM1 null genotypes (OR 1.18, 95% CI 0.97, 1.13) and increased risk of asthma; No evidence for GSTT1 and GSTP1.
Carroll et al. (2005) Dai et al. (2021)	Lung function	Adults and children	In adults, no evidence for GSTM1; in children, GSTM1 null and GSTP1 Val105 were associated with higher FEV1 and FVC.
Gilliland et al. (2002) Dai et al. (2018)	Parent-reported wheezing	Children	No evidence for GSTM1. GSTT1 and GSTP1.
Kabesch et al. (2004) Malling et al. (2012)	Parent-reported asthma	Children	No evidence for GSTM1 and GSTT1.

The most studied GST gene in relation to lung function and asthma outcomes is *GSTP1*, but the evidence for *GSTP1* risk alleles is inconsistent. As *GSTP1* with Ile/Ile genotypes has the highest enzyme activity, carriers of *GSTP1* with any Val alleles would be expected to have a higher risk of asthma symptoms and poorer lung function. There is some support for this hypothesis. A case control study in Turkey found that subjects with *GSTP1* homozygous Val/Val genotypes had a 3.55 fold increased risk of atopic asthma in adulthood (95%CI 1.10, 12.56) compared to those with homozygous Ile/Ile genotypes (Tamer et al., 2004). A United States cohort study over a 4-year period found that children with *GSTP1* Val/Val genotypes had slower lung function growth for FEV_1_ (-0.34%, 95% CI -0.68, 0) and FVC (-0.35%, 95% CI -0.50, -0.04) compared to those with any Ile genotypes (Gilliland et al., 2002). However, some studies have found the opposite relationship or null associations. A United Kingdom study conducted on adults reported that having the *GSTP1* homozygous Val variant was associated with a six-fold lower risk of asthma compared with homozygous Ile carriers (Anthony A et al., 2000). Moreover, there are several studies that have not found any association between the *GSTP1* genotype and respiratory health (Chung, 2002; Safronova et al., 2003; Nickel et al., 2005; Oh et al., 2005; Renate et al., 2005; Ercan et al., 2006).

People with *GSTM1*/*GSTT1* null genotypes have complete loss of enzyme activity resulting in a reduction of antioxidant capacity (Minelli et al., 2011). Carriers of *GSTM1* and *GSTT1* null genotypes may be more susceptible to asthma. Several case control studies have found a higher prevalence of both *GSTM1* null genotypes and *GSTT1* null genotypes in asthma cases, compared with controls (Vavilin et al., 2002; Mostafa et al., 2004; Zhang et al., 2004; Hanene et al., 2007; Karam et al., 2012; Iorio et al., 2015). However, these were all small studies with sample sizes of less than 300 participants. In contrast, two large cohort studies, the Swiss Study on Air Pollution and Lung Diseases in Adults (SAPALDIA) (Imboden et al., 2008) (*n* = 4,422), and the Avon Longitudinal Study of Parents and Children (ALSPAC) (Minelli et al., 2010) in the United Kingdom (*n* = 7,262), did not find evidence of associations between *GSTM1* or *GSTT1* genotypes and asthma.

Two systematic reviews have been conducted on the associations between *GSTM1*/T1/P1 genotypes and asthma (Minelli et al., 2010; Piacentini et al., 2013). One found weak evidence for an association between *GSTT1* null genotypes and increased risk of asthma (pooled OR 1.33, 95%CI 1.10,1.60), however, this study identified high heterogeneity in their analyses (Piacentini et al., 2013). There was little evidence, from these 2 reviews, to support a substantial independent role of GST genes in the development of asthma (Minelli et al., 2010; Piacentini et al., 2013). One hypothesis that may explain these findings is that *GSTM1*, *GSTT1* and *GSTP1* have overlapping substrate specificities, so that a deficiency in one isoform may be compensated for by another. One of the meta-analyses from these reviews included 14 additional studies to assess whether the combination of multiple GST risk genotypes may have an additive effect on respiratory health outcomes. This review found a higher risk of asthma for people with both *GSTM1* null and *GSTT1* null genotypes (OR 2.15, 95%CI 1.21, 3.71) compared to those with two non-null genotypes, but no evidence of an association in those with only one null *GSTM1* or *GSTT1* gene (Minelli et al., 2010). Other than GST genes, there are many antioxidants that may interact to impact the response to oxidative stress. One study investigated a dose-response relationship between *GSTM1*, *GSTP1* genotypes, another respiratory inflammatory biomarker, Clara Cell secretory protein-16, (CC16) and asthma risk. Individuals with three putative high-risk genotypes had the highest risk of asthma (OR: 22.5, 95% CI 4.08, 123.48), followed by moderately increased risk for two putative high-risk genotypes (OR: 5.50, 95% CI 1.96, 15.41) and little evidence for one putative high-risk genotype (OR: 2.05, 95% CI 0.77, 5.41) (Mostafa et al., 2004).

Studies of GST genes greatly improve the understanding of the pathophysiological mechanisms that may lead to asthma and lung function deficits. Current studies have unexpectedly shown relatively small associations between GST genes alone and these medical conditions. Apart from this being because of capacity through several antioxidant pathways as indicated above, this may also be due to individual differences in exposure to environmental factors. Many household air exposures like Environmental Tobacco Smoke (ETS) have been shown to induce oxidative stress, however, individuals differ in their ability to deal with an oxidant burden, and these differences may be partially due to GST genetic variation. GST genes, regulating antioxidant defences, are likely to modulate the response to pollutants. Thus, the effects of environmental exposures on respiratory health may be found only for individuals with a high-risk genetic profile who are also exposed to a high-risk environment. This hypothesis has been investigated in several studies as outlined below.

## Glutathione S-transferase genes, household oxidative exposures and asthma and lung function

### Evidence for *GSTM1* polymorphism interactions

#### Asthma

Evidence for an interactive effect of *GSTM1* on the association between household exposures and adverse respiratory outcomes is summarized in Table 3. The earliest studies that investigated *GSTM1* interactions in United States in 2002, suggested *in-utero* exposure to maternal smoking was associated with an increased risk of childhood asthma and wheezing occurrence at school age, but only for children with *GSTM1* null genotypes (Interaction *p* values <0.05). In this study, neither the *GSTM1* genotype, nor *in-utero* exposure to smoking were individually associated with any asthma or wheezing outcomes (Frank D et al., 2002). Subsequently, similar studies have also identified significant interactions between household air pollution, *GSTM1* null genotypes and asthma risk (Carr et al., 2004; Wu et al., 2014). In contrast, the evidence from some studies has not supported this *GSTM1* interaction (Lee et al., 2007; Wang et al., 2011; Hersoug et al., 2012; Dai et al., 2019; Dai et al., 2021). The reasons are not clear, but the interaction may be exposure specific, with studies investigating tobacco smoke exposure more likely to find adverse associations when compared to other household air pollution exposures (Dai et al., 2018). Studies that investigated incense burning (Wang et al., 2011) and wood stove/candles during wintertime (Hersoug et al., 2012) found no evidence of interactions.

**TABLE 3:** Influence of GSTM1 polymorphisms on the relationship between household environmental exposures and asthma and lung function.

References	Study design	Household exposures	Respiratory outcomes	Outcome age	Results (interaction)	Risk alleles
Gilliland et al. (2002) Dai et al. (2018)	Cross sectional	*In-utero* maternal smoking	Ever/current asthma, medication for asthma, early onset asthma, persistent asthma, ever wheezing, wheeze with/without cold, persistent wheeze.	Over 8 years (range 8–14 years)	Evidence for GSTM1 interaction (*p* < 0.05 for a range of asthma and wheezing outcomes).	GSTM1 Null
He et al. (2002) Palmer et al. (2006a)	Case control nested in a cohort	Active smoking during last 5 years	FEV_1_ %predicted	35–60 years	No evidence of interaction	N/A
He et al. (2004) Rogers et al. (2009)	Case control nested in a cohort	Smoking history (pack-year)	High lung function and low lung function group	35–60 years	No evidence of interaction	N/A
Kabesch et al. (2004) Malling et al. (2012)	Cross sectional	*In-utero* /current ETS	Current/ever wheeze, current wheeze.	9–11 years	Evidence for GSTM1 interaction (*p* values ranged from (0.05–0.77).	GSTM1 Null
Palmer et al. (2006a) Owens et al. (2019)	Cohort	ETS	PEFR %predicted, FEV_1_, and FVC	2 groups: 3–12 years and 13–21 years	Evidence of interaction in older asthmatic children.	GSTM1 Null
Imboden et al. (2007) Breton et al. (2009)	Cohort	Ever smoking/never smoker/persistent smoker	Difference in mean annual change in FEV_1_, FVC and FEF_2575_ over 11 years	Age at baseline 40.8 ± 11.5 years	No evidence of interaction.	N/A
Lee et al. (2007) Kabesch et al. (2004)	Case control	Household ETS	Ever/current wheeze	Cases: 11.8 ±1.7 years	No evidence of interaction.	N/A
				Controls: 12.1 ± 1.8 years		
Li et al. (2009) He et al. (2004)	Cross sectional	ETS	Ever asthma	Grade 1–6	Borderline evidence for GSTM1 interaction (no *p* value reported)	GSTM1 present
Murdzoska et al. (2009) Li et al. (2008)	Cohort	*In-utero* smoking	Maximal flow at functional residual capacity (Vmax_FRC_)	1, 6 and 12 months	No evidence of interaction.	N/A
Rogers et al. (2009) Panasevich et al. (2010)	Cohort	*In-utero* and postnatal ETS	Current asthma, FEV_1_ %predicted, FVC %predicted, FEV_1_/FVC	8.8 years ± 2.1	Evidence of GSTM1 interaction for *in-utero* (*p* = 0.02) and ETS (*p* = 0.08) for asthma outcomes, and *p* < 0.05 for lung function.	GSTM1 Null
Wang et al. (2011) Wang et al. (2010)	Cross sectional	Incense burning smoke in past 12 months	Ever/current asthma, ever/current wheeze, use of asthma medication, exercise wheeze	12.26 ±0.50 years	No evidence of interaction.	N/A
Hersoug et al. (2012) Lee et al. (2015)	Cross sectional	ETS, use of wood stove/candles during wintertime, active smoking	Current wheeze, FEV1 %predicted	18–69 years	No evidence of interaction (*p* values ranged from 0.3 to 0.9).	N/A
Malling et al. (2012) Schroer et al. (2009)	Cross sectional	Current active smoker/ever smoker	FEV_1_ %predicted, FEV_1_/height^2^	22–44 years	Evidence of GSTM1 interaction (*p* = 0.03).	GSTM1 Null
Wu et al. (2014) Gerbase et al. (2011)	Cohort	*In-utero*/family member smoked	Ever asthma	6 years	Evidence of interaction for ever asthma in girls. No associations found for dermatitis and rhinitis.	GSTM1 Null
Dai et al. (2019) Munoz et al. (2012)	Cohort	Perinatal smoking	Asthma, FEV1, FVC	12 and 18 years	Evidence of interaction on FEV_1_ and FVC at 18 years. No evidence at 12 years and at asthma outcomes.	GSTM1 Null
Owens et al. (2019) Palmer et al. (2006b)	Cohort	*In-utero* smoking	Change in FEV_1_ %predicted and FVC %predicted	Between 6–24 years	Evidence of interaction of FEV1 and FVC %predicted (no *p* value provided)	GSTM1 Null
Dai et al. (2021) Dai et al. (2016)	Cohort	Household air pollution	Asthma, changes in FEV1, FVC, FEV1/FVC	Between 43–53 years	Evidence of interaction on FEV_1_/FVC, no evidence on asthma.	GSTM1 Null

#### Lung function

Several studies have investigated interactions between *GSTM1* polymorphisms and environmental exposures on the risk of lung function deficits. Overall, the evidence supports a relationship between *GSTM1* null genotypes and increased risk of lung function deficits in individuals exposed to different kinds of household air pollution. Three studies found that carriers of *GSTM1* null variants, who were exposed to smoking, had an increased risk of impaired lung function in terms of reduced FEV_1_ %predicted (Palmer et al., 2006a; Malling et al., 2012), FVC %predicted and FEV_1_/FVC ratio compared to those with *GSTM1* present variants (Rogers et al., 2009). Recent studies have focused on lung function changes over a longer period, and have identified both reduced lung function growth (Owens et al., 2019) and increased lung function decline (Dai et al., 2021) for exposed participants with *GSTM1* null genotypes. There appears to be more evidence for this interaction when measuring lung function as opposed to asthma even in the same participants. This may be partly explained by lung function being a continuous variable, with greater power to be detect associations when compared to categorized outcomes (asthma).

### Evidence for *GSTT1* polymorphism interactions

#### Asthma

The evidence regarding an interactive effect of *GSTT1* on the relationship between indoor exposures and adverse respiratory outcomes is presented in Table 4. Overall, there was less evidence for an interaction in relation to asthma outcomes (compared to *GSTM1*). There were only two cross-sectional studies that investigated asthma and both found significant interactions for *GSTT1* in school aged children. One study found that the frequency of incense burning was associated with an increased risk of current asthma, asthma medication use, lifetime wheeze, nocturnal wheeze, and exercise wheeze in children with *GSTT1* null genotypes. These associations were not seen in those with *GSTT1* present genotypes (Wang et al., 2011). The second study also found evidence that *GSTT1* null children had an increased risk of ever wheeze with ETS exposure, compared with *GSTT1* present children not exposed to ETS. There were no associations found for GST present children with ETS exposure and GST null children without ETS exposure (Kabesch et al., 2004). A further study from Copenhagen considered an expanded number of common indoor air pollutants like the use of woodstoves and candles on wheeze outcomes, however, no evidence of an interaction was found (all interactions *p* > 0.2) (Hersoug et al., 2012).

**TABLE 4:** Influence of GSTT1 polymorphisms on the relationship between household environmental exposures and asthma and lung function.

References	Study design	Household exposures	Respiratory outcomes	Outcome age	Results (interaction)	Risk alleles
He et al. (2002) (Palmer et al., 2006a)	Case control nested in a cohort	Active smoking during last 5 years	FEV_1_ %predicted	35–60 years	No evidence of interaction.	N/A
He et al. (2004) (Rogers et al., 2009)	Case control nested in a cohort	Smoking history (pack-year)	High lung function and low lung function group	35–60 years	Evidence of GSTT1 interaction (*p* = 0.038)	GSTT1 Null
Kabesch et al. (2004) Malling et al. (2012)	Cross sectional	*In-utero* /current ETS	Current/ever wheeze, current wheeze.	9–11 years	Evidence for GSTT1 interaction (*p* values ranged from (0.05–0.77).	GSTT1 Null
Imboden et al. (2007) Breton et al., (2009)	Cohort	Ever smoking/never smoker/persistent smoker	Difference in mean annual change in FEV_1_, FVC and FEF_2575_ over 11 years	Age at baseline 40.8 ± 11.5 years	In male, evidence of GSTT1 interaction on FEV_1_ decline in ever smoker (*p* < 0.001) and persistent smoker (0.029).	GSTT1 Null
Murdzoska et al. (2009) Li et al. (2008)	Cohort	*In-utero* smoking	Maximal flow at functional residual capacity (Vmax_FRC_)	1, 6 and 12 months	Evidence of GSTT1 interaction (*p* = 0.008)	GSTT1 Null
Wang et al. (2011) Wang et al. (2010)	Cross sectional	Incense burning smoke in past 12 months	Ever/current asthma, ever/current wheeze, use of asthma medication, exercise wheeze	12.26 ±0.50 years	Evidence of GSTT1 interaction (*p* < 0.05).	GSTT1 Null
Hersoug et al. (2012) Lee et al. (2015)	Cross sectional	ETS, use of wood stove/candles during wintertime, active smoking	Current wheeze, FEV_1_ %predicted	18–69 years	No evidence of interaction (*p* values ranged from 0.3 to 0.9).	N/A
Malling et al. (2012) Schroer et al. (2009)	Cross sectional	Current active smoker/ever smoker	FEV_1_ %predicted, FEV_1_/height^2^	22–44 years	No evidence of interaction.	N/A
Dai et al. (2019) Munoz et al. (2012)	Cohort	Perinatal smoking	Asthma, FEV1, FVC	12 and 18 years	Evidence of interaction on FEV_1_ and FVC at both 12 and 18 years, no evidence on asthma.	GSTT1 Null
Owens et al. (2019) Palmer et al. (2006b)	Cohort	*In-utero* smoking	Change in FEV_1_ %predicted and FVC %predicted	Between 6–24 years	No Evidence of interaction (No *p* value provided)	N/A
Dai et al. (2021) Dai et al. (2016)	Cohort	Household air pollution	Asthma, changes in FEV1, FVC, FEV_1_/FVC	Between 43–53 years	Evidence of interaction on FEV_1_, FVC, and FEV1/FVC, no evidence on asthma.	GSTT1 Null

#### Lung function

There was more evidence for interaction on lung function compared with asthma. One study found that amongst infants exposed to tobacco smoke *in utero*, those with *GSTT1* present had increased VFRCmax in the first year of life compared with *GSTT1* null infants (interaction *p* = 0.037). No evidence of association was seen in the groups (*GSTT1* present and absent) not exposed to *in utero* tobacco smoke (Murdzoska et al., 2010). Household smoking may also influence lung function changes over a longer term for those with *GSTT1* null genotypes (Imboden et al., 2007; Dai et al., 2019; Dai et al., 2021). In contrast, another two cross-sectional studies conducted on adults that investigated current household air pollution exposure, found no evidence of an interaction (He et al., 2004; Imboden et al., 2007).

### Evidence for *GSTP1* polymorphism interactions

#### Asthma

Evidence for a potential interactive effect of *GSTP1* on the relationship between indoor exposures and adverse respiratory outcomes is presented in Table 5. Although *GSTP1* is the most studied of the three mentioned GST polymorphisms, particularly in conjunction with more varied exposures (different forms of indoor air pollution), the findings with respect to risk alleles are more inconsistent. Some studies have provided evidence that *GSTP1* with Val alleles is associated with increased risk of asthma/wheeze outcomes in childhood with exposure to *in-utero* smoking (Lee et al., 2007; Li et al., 2008; Panasevich et al., 2010; Wang et al., 2010). Another study found evidence for a three-way interaction in school-aged children with *GSTP1* homozygous Ile genotypes and low vitamin A levels who had an increased risk of asthma diagnosis (OR 4.44, 95% CI 1.58, 12.52) compared to children who did not have these two risk factors (Lee et al., 2015). Other studies have found no evidence of an interaction between *GSTP1* genotypes and indoor air exposures (Schroer et al., 2009; Gerbase et al., 2011; Munoz et al., 2012) and asthma. Recently, we performed a latent class analysis combining several household air pollution sources into exposure profiles. Our study found that profiles of household air pollution exposure were associated with increased risk of asthma in middle-aged adults with *GSTP1* Ile/Ile genotypes; however, the associations were not found in participants with other *GSTP1* genotypes (Dai et al., 2021). Though this study was again limited by small sample size, multiple exposure profiles were associated with higher respiratory risk than the individual household exposures (Dai et al., 2021). Not considering household air pollution exposures as complex mixtures or exposure phenotypes may be one reason for the lack of current evidence of an association between household air pollution (excluding smoking) and lung health outcomes.

**TABLE 5:** Influence of GSTP1 polymorphisms on the relationship between household environmental exposures and asthma and lung function.

References	Study design	Household exposures	Respiratory outcomes	Outcome age	Results (interaction)	Risk alleles
He et al. (2002) Palmer et al. (2006a)	Case control nested in a cohort	Active smoking during last 5 years	FEV_1_ %predicted	35–60 years	No evidence of interaction.	N/A
He et al. (2004) (Rogers et al., 2009)	Case control nested in a cohort	Smoking history (pack-year)	High lung function and low lung function group	35–60 years	No evidence of interaction	N/A
Imboden et al. (2007) Breton et al. (2009)	Cohort	Ever smoking/never smoker/persistent smoker	Difference in mean annual change in FEV_1_, FVC and FEF_2575_ over 11 years	Age at baseline 40.8 ± 11.5 years	No evidence of interaction.	N/A
Lee et al. (2007) Kabesch et al. (2004)	Case control	Household ETS	Ever/current wheeze	Cases: 11.8 ±1.7 years	Evidence of GSTP1 interaction (*p* = 0.01 for ever wheeze, *p* = 0.004 for current wheeze).	GSTP1 with any Val allele
				Controls: 12.1 ± 1.8 years		
Li et al. (2008) (57)	Cross sectional	*In-utero* smoking	Ever asthma, current wheeze, early/late onset asthma, medication for wheeze	8–18 years	Evidence for interaction on outcome current wheeze (*p* = 0.035), and medication for wheezing (*p* = 0.043).	GSTP1 with any Val allele
Murdzoska et al. (2009) Li et al. (2008)	Cohort	*In-utero* smoking	Maximal flow at functional residual capacity (VFRCmax)	1, 6 and 12 months	No evidence of interaction.	N/A
Schroer et al. (2009) Minelli et al. (2010)	Cohort	Household ETS	Wheezing at 12 and 24 months, persistent wheezing	12 and 24 months	No evidence of interaction (*p* > 0.2).	N/A
Panasevich et al. (2010) (58)	Cohort	Early life maternal smoking (pregnancy and early childhood)	Current asthma, early/late onset wheeze, transient wheeze	4 years	Insignificant evidence of GSTP1 on early onset wheeze (*p* = 0.17).	GSTP1 with any Val allele
Schultz et al. (2010) (59)	Cross sectional	ETS	Asthma severity,	2–16 years	Evidence of interaction on atopy, not asthma severity.	GSTP1 with Ile/Ile
Wang et al. (2011) Wang et al. (2010)	Cross sectional	Incense burning smoke in past 12 months	Ever/current asthma, ever/current wheeze, use of asthma medication, exercise wheeze	12.26 ±0.50 years	No evidence of interaction.	N/A
Malling et al. (2012) Schroer et al. (2009)	Cross sectional	Current active smoker/ever smoker	FEV_1_ %predicted, FEV_1_/height^2^	22–44 years	No evidence of interaction.	N/A
Munoz et al. (2012) (60)	Case control	ETS	Asthma (no clear definition)	6–9 years	No evidence of Interactions	N/A
Lee et al. (2015) (61)	Cross sectional	ETS	Current asthma	7–12 years	No evidence of GSTP1 interaction, but 3-way interaction were seen for those with Ile/Ile genotype for exposed children plus low vitamin A.	GSTP1 Ile/Ile
Dai et al. (2019) Munoz et al. (2012)	Cohort	Perinatal smoking	Asthma, FEV_1_, FVC	12 and 18 years	Evidence of interaction on FEV_1_ and FVC at 18 years. No evidence at 12 years and at asthma outcomes.	GSTP1 Ile/Ile
Dai et al. (2021) Dai et al. (2016)	Cohort	Household air pollution	Asthma, changes in FEV1, FVC, FEV1/FVC	43–53 years	Evidence of interaction on FEV_1_, FVC, FEV_1_/FVC and asthma.	GSTP1 Ile/Ile

#### Lung function

Several studies have focussed on three-way interactions to take age into account. One childhood study provided evidence on the potential adverse role of *GSTP1* val105 on lung function when exposed to second-hand tobacco smoke. This association was found only in older children (13–21 years vs 3–12 years) (Palmer et al., 2006b). The results were consistent with our study demonstrating that an interaction of *GSTP1* with lung function was only found in participants at 18 years, not at 12 years (Dai et al., 2019). However, this study found that adolescents with *GSTP1* Ile/Ile genotypes were more likely to have lower lung function (Dai et al., 2019). Gender is also a potential third factor to be considered in *GSTP1* interactions. A Swiss study found that heterozygosity for *GSTP1* (heterozygous Ile105Val) was associated with slower decline in FVC in female persistent smokers compared to those with homozygous Ile genotypes, while no association was observed in the female never-smokers’ group (Imboden et al., 2007). However, female smokers with *GSTP1* with homozygous Val genotypes did not show an increased decline compared to those with a homozygous Ile genotype. These associations were not seen in males (Imboden et al., 2007).

## Reasons for inconsistent findings

This review has provided evidence that GST genes may modify the link between household air pollution exposures and asthma and lung function. However, the associations differ depending on exposure time, types, risk alleles, and study design. There was evidence that GST gene interactions were more consistent for studies conducted on children, suggesting that the effect of gene-pollution interactions on respiratory health may vary by age. There are many differences between children and adults regarding respiratory system pathophysiology and immune responses. Children are relatively naïve with respect to many body systems and may be more susceptible to noxious exposures. Consequently, oxidative capacity may be more important for children compared to adults. Furthermore, the exposures documented in childhood studies usually occur during early life when the lungs grow. Airways, alveoli and blood vessels grow rapidly up to 2 years of age and continue to expand until early adulthood, and exposures during this critical period have been found to detrimentally affect subsequent respiratory health (Dai et al., 2016). As such, my review also supports the notion that there are critical time windows for adverse exposures and the impact is higher during lung growth in early life. Asthma and lung function deficits are both conditions that develop over an extended period. Longitudinal cohorts provide better evidence than cross-sectional and case control studies for investigating these chronic conditions and, are more likely to detect associations, particularly for lung function outcomes.

Though GSTs play an important role in regulating oxidative stress, many other factors are also involved in supporting an antioxidative stress network to protect respiratory health. There are several antioxidant genes that have been identified as potentially important for lung health and have been studied including: NAD(P)H dehydrogenase [quinine] 1 (NQO1) and heme oxygenase 1 (HMOX-1) (Minelli et al., 2011). The effect of each antioxidant gene individually may be very small, hard to detect and dependent on the presence of specific environmental exposures. However, multiple interactions may alter individual susceptibility to the effects of air pollution on respiratory health (Minelli et al., 2011). One study suggested that supplemental nutrition intake, like vitamin A, could enhance antioxidant capacity and possibly prevent respiratory harm in susceptible children with risk GST genotypes exposed to ETS (Lee et al., 2015). In future, clinicians could consider antioxidant supplements as additions to personalised therapy for children found to have a high-risk genotype on genetic screening.

Findings from this review differed in terms of the specific risk alleles and exposures. This was particularly the case for *GSTP1* risk alleles where the evidence was contradictory and inconsistent. It is difficult to draw a firm conclusion on *GSTP1* risk alleles based on current evidence. One of the reasons for this lack of consistency could be that this polymorphism was investigated using Single Nucleotide Polymorphisms (SNPs), not haplotypes. *GSTP1* has a more complex genetic variation than *GSTM1* and *GSTT1*, and has recognized haplotypes, in terms of 4 alleles: GSTP1*A (Ile105-Ala114), GSTP1*B (Val105-Ala114), GSTP1*C (Val105-Val114) and GSTP1*D (Ile105-Val114). The included papers have generally focussed on the Ile105-Val105 substitution because the minor Ala114Val allele has a lower frequency and many of the studies had small numbers so were unable to address *GSTP1* using these 4 distinct haplotypes. Most studies of *GSTP1* interaction did not take into account the effect of the Ala114Val allele. However, Ala114Val may interact with Ile105Val alleles to produce heterogeneity in different scenarios [262]. Future studies investigating more comprehensive genetic variation and haplotypes of the *GSTP1* genes would improve our understanding of the role of *GSTP1* on respiratory health.

## Conclusion

This review provides evidence for the presence of gene-environment interactions and highlights the potential biological mechanisms relating to the role of oxidative stress in the development and progression of respiratory disease. Many household air pollution exposures may have health effects for susceptible populations; for individuals with particular GST genotypes. Further studies should consider other exposures that may interact with GST genes to facilitate the oxidative stress pathway including nutrition, and physical activity. Personalised strategies may identify high risk people and help in targeted prevention of respiratory disease.
